# Change in iron metabolism in rats after renal ischemia/reperfusion injury

**DOI:** 10.1371/journal.pone.0175945

**Published:** 2017-04-20

**Authors:** Guang-liang Xie, Lin Zhu, Yan-min Zhang, Qian-nan Zhang, Qing Yu

**Affiliations:** Department of Nephrology, Shanghai General Hospital, Shanghai Jiaotong University School of Medicine, Shanghai, P. R. China; Lady Davis Institute for Medical Research, CANADA

## Abstract

Previous studies have indicated that hepcidin, which can regulate iron efflux by binding to ferroportin-1 (FPN1) and inducing its internalization and degradation, acts as the critical factor in the regulation of iron metabolism. However, it is unknown whether hepcidin is involved in acute renal ischemia/reperfusion injury (IRI). In this study, an IRI rat model was established via right renal excision and blood interruption for 45 min in the left kidney, and iron metabolism indexes were examined to investigate the change in iron metabolism and to analyze the role of hepcidin during IRI. From 1 to 24 h after renal reperfusion, serum creatinine and blood urea nitrogen were found to be time-dependently increased with different degrees of kidney injury. Regular variations in iron metabolism indexes in the blood and kidneys were observed in renal IRI. Renal iron content, serum iron and serum ferritin increased early after reperfusion and then declined. Hepcidin expression in the liver significantly increased early after reperfusion, and its serum concentration increased beginning at 8 h after reperfusion. The splenic iron content decreased significantly in the early stage after reperfusion and then increased time-dependently with increasing reperfusion time, and the hepatic iron content showed a decrease in the early stage after reperfusion. The early decrease of the splenic iron content and hepatic iron content might indicate their contribution to the increase in serum iron in renal IRI. In addition, the duodenal iron content showed time-dependently decreased since 12 h after reperfusion in the IRI groups compared to the control group. Along with the spleen, the duodenum might contribute to the decrease in serum iron in the later stage after reperfusion. The changes in iron metabolism indexes observed in our study demonstrate an iron metabolism disorder in renal IRI, and hepcidin might be involved in maintaining iron homeostasis in renal IRI. These findings might suggest a self-protection mechanism regulating iron homeostasis in IRI and provide a new perspective on iron metabolism in attenuating renal IRI.

## Introduction

Ischemia/reperfusion injury (IRI) is a common pathogenesis of acute kidney injury (AKI) because the kidneys are highly perfused organs and are very sensitive to ischemia. Many procedures and factors can result in renal IRI, such as renal vascular surgery, kidney transplant, cardiac arrest, hypotension and shock [1] IRI is a pathological phenomenon in which renal tissue and cell damage are caused by ischemic aggravation after blood reperfusion, which is a key factor leading to renal failure and a poor prognosis. Therefore, it is of great importance to study the underlying pathological mechanism and reaction mechanism of the body, exploring potential prevention and treatment strategies for IRI. Kidneys function in iron filtration and reabsorption [2], which are important in iron homeostasis. However, studies have shown that iron itself and iron-related oxidative stress may play important roles in kidney injury under pathologic conditions [3]. Ferrous ions have direct cytotoxic effects, especially to proximal tubular epithelial cells [4]. Additionally, iron can catalyze the generation of hydroxyl free radicals and lipid peroxidation, leading to oxidative stress and tissue damage [5]. These findings suggest that iron metabolism disorder may occur during IRI.

In recent years, the discovery of hepcidin provided a new way to study the mechanism of iron metabolism. Many studies suggest that hepcidin is mainly synthesized and secreted by hepatocytes [6, 7]. It regulates iron efflux by binding to ferroportin-1 (FPN1), inducing its internalization and degradation, and it acts as the critical factor in regulating iron homeostasis [8, 9]. At present, the relationship between hepcidin and chronic kidney disease-related anemia has been intensively studied in animals and humans [10–12]. However, whether hepcidin is involved in acute renal IRI is unclear.

In this study, we aimed to investigate the possible changes in hepcidin and iron metabolism indexes to reveal the relationship between renal IRI and these elements. We are also interested in whether or not there is a possible regulatory mechanism of iron homeostasis during renal IRI.

## Materials and methods

### Ethics statement and animals

This study was approved by the Institutional Animal Care and Use Committee of Shanghai Jiaotong University. The procedures were carried out according to the National Institute of Health guidelines. A total of 48 healthy adult Sprague-Dawley (Sino-British SIPPR/BKLab. Animal Ltd., Co., Shanghai, China) male rats (weighing 200±20 g) were used in this study. Prior to the experiment, all rats were allowed free access to standard diet and water and were subjected to a circadian rhythm with a 12 h day and 12 h night at an ambient temperature of 24~26°C with 50~60% humidity for 1 week. The rats were randomly divided into eight groups including a control group (n = 6) and a renal IRI model group (n = 42) that was subsequently divided into seven groups (n = 6 each group) based on reperfusion time: IRI 1 h, 4 h, 8 h, 12 h, 16 h, 20 h, and 24 h.

### Animal model of renal IRI

Renal ischemia/reperfusion (IR) was performed in Sprague-Dawley rats as previously described [13]. Briefly, rats were anesthetized with an intraperitoneal injection of sodium pentobarbital at a dose of 40 mg/kg (Sigma, St. Louis, USA) and then placed in a supine position. The kidneys were accessed through a 2.5-cm midline abdominal incision. The right kidney was then removed, and a non-traumatic vascular clamp (FST, Essen, Germany) was applied to the left renal pedicle for 45 min. Then, the clamp was removed for renal reperfusion. Occlusion was verified visually based on a change in the color of the kidneys to an atropurpureus shade, and reperfusion was verified visually based on a change in the color of the kidneys to a blush tone. Finally, the abdominal cavity was closed. The animals in the control group underwent right kidney excision without clamping of the left renal pedicles. Samples were collected at 1 h, 4 h, 8 h, 12 h, 16 h, 20 h and 24 h after renal reperfusion in the IRI groups and at 1 h after a 45-min exposure of the left kidney in the control group. Once the rats were under anesthesia, blood samples were collected via venipuncture from the postcava into vacuum tubes without anticoagulant. Then, the blood samples were centrifuged, and the serum was stored at -80°C for further analysis. Parts of the liver and kidneys were fixed in 10% buffered formalin for histological examination. The rest of the liver and kidneys, as well as the spleen and duodenum, were frozen in liquid nitrogen and stored at -80°C for further analysis.

### Determination of blood biochemistry and iron metabolism indexes in the serum

The levels of serum creatinine (SCr), blood urea nitrogen (BUN), serum iron (SI), and serum ferritin (SF) were determined using an automated Biochemical Analyzer (Beckman Coulter, Inc., CA, USA). Hepcidin levels in serum samples were quantified using an enzyme-linked immunosorbent assay (ELISA) (Jiancheng Biotechnology, Nanjing, China) according to the manufacturer’s instructions.

### Iron content analysis

Samples of liver, kidney, spleen and duodenum were respectively ground with normal saline to make tissue homogenates. After microcentrifugation (Eppendorf, Germany) (2500 *g* for 10 min at 4°C), supernatants of tissues were collected, and protein concentrations were determined using a bicinchoninic acid (BCA) protein assay kit (Beyotime, Nanjing, China). According to the iron content assay kit instructions (Jiancheng Biotechnology, Nanjing, China), the iron contents of tissue homogenates were determined spectrophotometrically by measuring the presence of iron bispyridine reactive substances. Absorbance was measured using a spectrophotometer (BIO-RAD, USA) at 520 nm. The results are expressed as μmol/g of protein based on a prepared standard graph.

### Histopathology

After fixation of the kidney samples that were placed in 10% formalin for 48 h, histological paraffin blocks were routinely prepared. A microtome (Leica Rotary; Leica Microsystems GmbH, Wetzlar, Germany) was used to obtain 4-mm-thick sections from the paraffin blocks. Collected sections were stained with hematoxylin-eosin (HE). Then, the sections were examined under 200x magnification using a light microscope (Leica DM5500B; Leica Microsystems GmbH, Wetzlar, Germany) by a pathologist blinded to the groups, and photos were taken.

### Real time RT-PCR

Total RNA was isolated from liver and kidney samples using Trizol reagent (Invitrogen, Carlsbad, CA, USA). A total of 1 μg of total RNA was transcribed into cDNA using PrimeScript RT master mix (Takara, Japan). All polymerase chain reactions (PCRs) were performed using an ABI ViiA^™^ 7 Real-Time PCR System (Applied Biosystems, ABI, USA) and SYBR green (Takara, Japan) in a total volume of 20 μL. β-Actin served as an internal control [14]. The following primer sequences were used:

hepcidin forward: 5’-TCTCCTGCTTCTCCTCCTG-3’

hepcidin reverse: 5’-TGTTATGCAACAGAGACCACA-3’

FPN1 forward: 5’-TTGCTGTTCTTTGCCTTAGTTGT-3’

FPN1 reverse: 5’-GAGGAGGCTGTTTCCGTAGAG-3’

β-actin forward: 5’-AGGATGCAGAAGGAGATTACTGC-3’

β-actin reverse: 5’-AAAACGCAGCTCAGTAACAGTGC-3’.

From each amplification plot, a threshold cycle (Ct) value and the number of transcripts were calculated. Relative mRNA levels were quantified using the equation 2^-(Ct sample—Ct beta-actin)^.

### Western blotting

Proteins from liver and kidney tissues were extracted using RIPA buffer with the inhibitor phenylmethanesulfonyl fluoride (Beyotime, Nanjing, China). Protein concentrations were determined using a BCA protein assay kit (Beyotime, Nanjing, China). A total of 40 μg of each protein was loaded into 10% SDS-PAGE gels and transferred to nitrocellulose membranes (Millipore, Billeria, USA). Then, the membranes were blocked for 2 h with 5% skim milk in TBST buffer. The blocked membranes were incubated with rabbit anti-hepcidin (1:500 dilution; Abcam, Cambridge, UK), rabbit anti-FPN1 (1:1000 dilution; Abcam, Cambridge, UK), and rabbit anti-β-actin (1:1000 dilution; Abcam, Cambridge, UK) antibodies at 4°C overnight. After incubation with a secondary antibody conjugated to horseradish peroxidase (1:1000, SantaCruz Biotechnoloy, CA, USA) for 1.5 h at room temperature, proteins were visualized using ECL reagents (Millipore, Billeria, USA).

### Immunohistochemistry

After deparaffinization and rehydration, paraffin sections of liver and kidney tissues were incubated with 3% hydrogen peroxide for 15 min to quench endogenous peroxidase activity. Then, the sections were subjected to antigen retrieval by heating the sections in a microwave oven in 10 mM sodium citrate buffer (pH 6.0). After blocking with 5% normal goat serum in PBS, liver tissue sections were incubated with rabbit anti-hepcidin (1:200 dilution; Abcam, Cambridge, UK) and kidney tissue sections were incubated with rabbit anti-FPN1 (1:400 dilution; Abcam, Cambridge, UK) at 4°C overnight. After washing with PBS, the sections were incubated with secondary antibodies. Finally, the sections were incubated with H_2_O_2_-DAB. Negative controls were incubated with 3% serum without primary antibodies. The integrated optical densities (IODs) of hepcidin and FPN1 were analyzed using Image-Pro Plus 6.0 software (Media Cybernetics, Bethesda, Maryland, USA).

### Statistical analysis

All data are expressed as the mean ± standard deviation. One-way analysis of variance (ANOVA) was performed for multi-group comparisons, t-test was performed between the control group and each IRI group. Statistical analysis was carried out using Statistical Package for Social Sciences (SPSS) (Version 19.0. Armonk, NY: IBM Corp. Chicargo, USA). A *P*-value of <0.05 was considered statistically significant.

## Results

### Histological and renal function analyses after renal IRI

**Fig 1:**
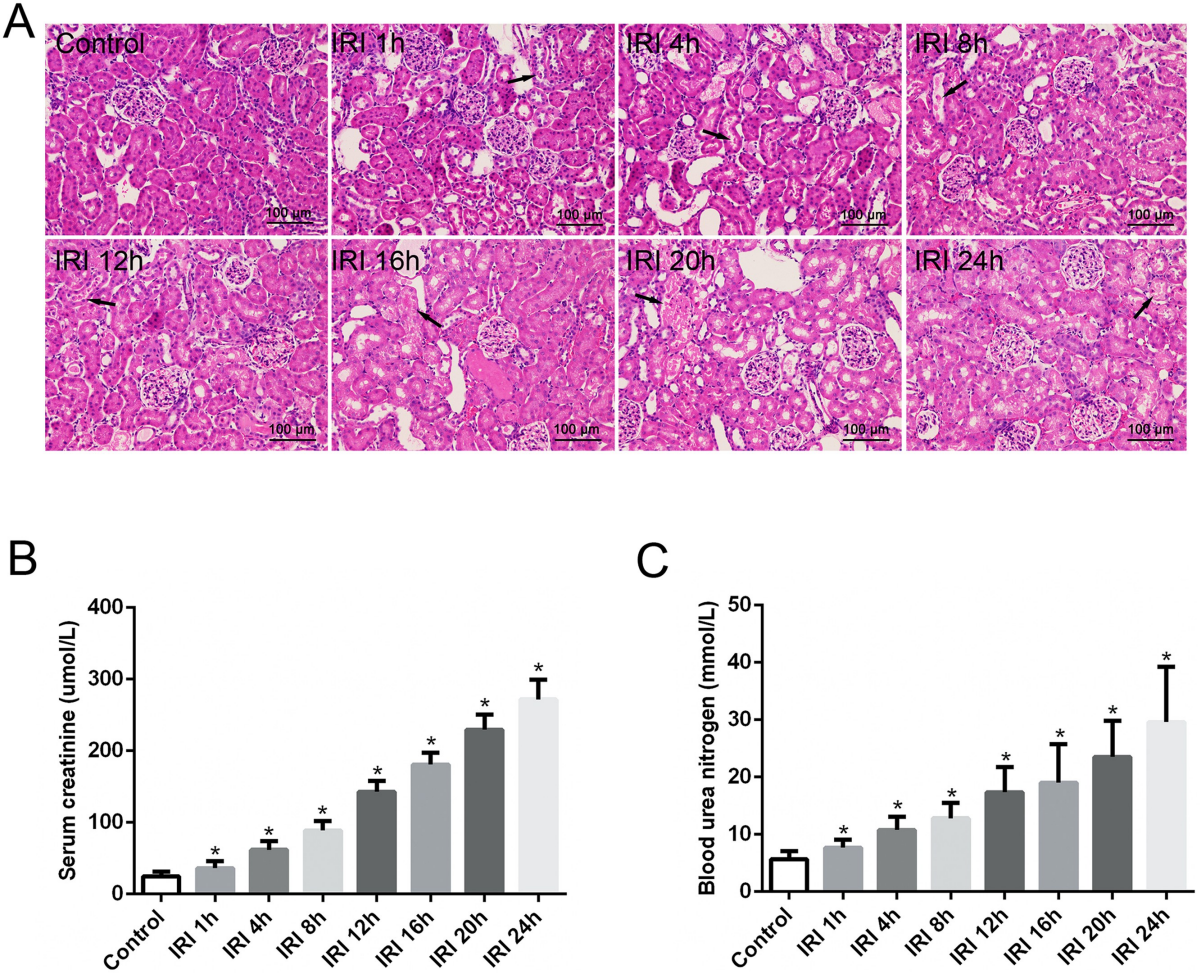
Renal pathological changes and changes in renal function after renal IRI. (A) Varying degrees of renal pathological changes from 1 h to 24 h after reperfusion in the IRI groups, including loss of brush borders, vacuolar degeneration and necrosis in epithelial cells, dilatation, cast formation, and cell debris in the kidney tubules. Arrow denotes pathological change of the renal tissue. The control group displayed normal histology. (B) SCr and (C) BUN levels were significantly elevated in the IRI groups compared with levels in the control group, and both SCr and BUN were time-dependently elevated from 1 h to 24 h after reperfusion in the IRI groups. Kidney sections were stained with hematoxylin and eosin. Original magnification, X200. The data are presented as the means ± standard deviation. * P <0.05, compared to control group; n = 6 in each group. IRI: ischemia/reperfusion injury.

Renal IRI was confirmed by analyzing the pathological changes of renal tissues and renal function after renal reperfusion. Analysis of the routinely HE-stained kidney tissue sections revealed varying degrees of renal pathological changes from 1 h to 24 h after reperfusion in the IRI groups, including the loss of brush borders, vacuolar degeneration and necrosis in the epithelial cells, as well as dilatation, cast formation, and cell debris in the kidney tubules, while the control group displayed normal histology (Fig 1A). The SCr and BUN levels were significantly elevated in the IRI groups compared with levels in the control group, and both SCr and BUN were time-dependently elevated from 1 h to 24 h after reperfusion in the IRI groups (Fig 1B and 1C).

### Evaluation of iron metabolism indexes in the serum after renal IRI

**Fig 2:**
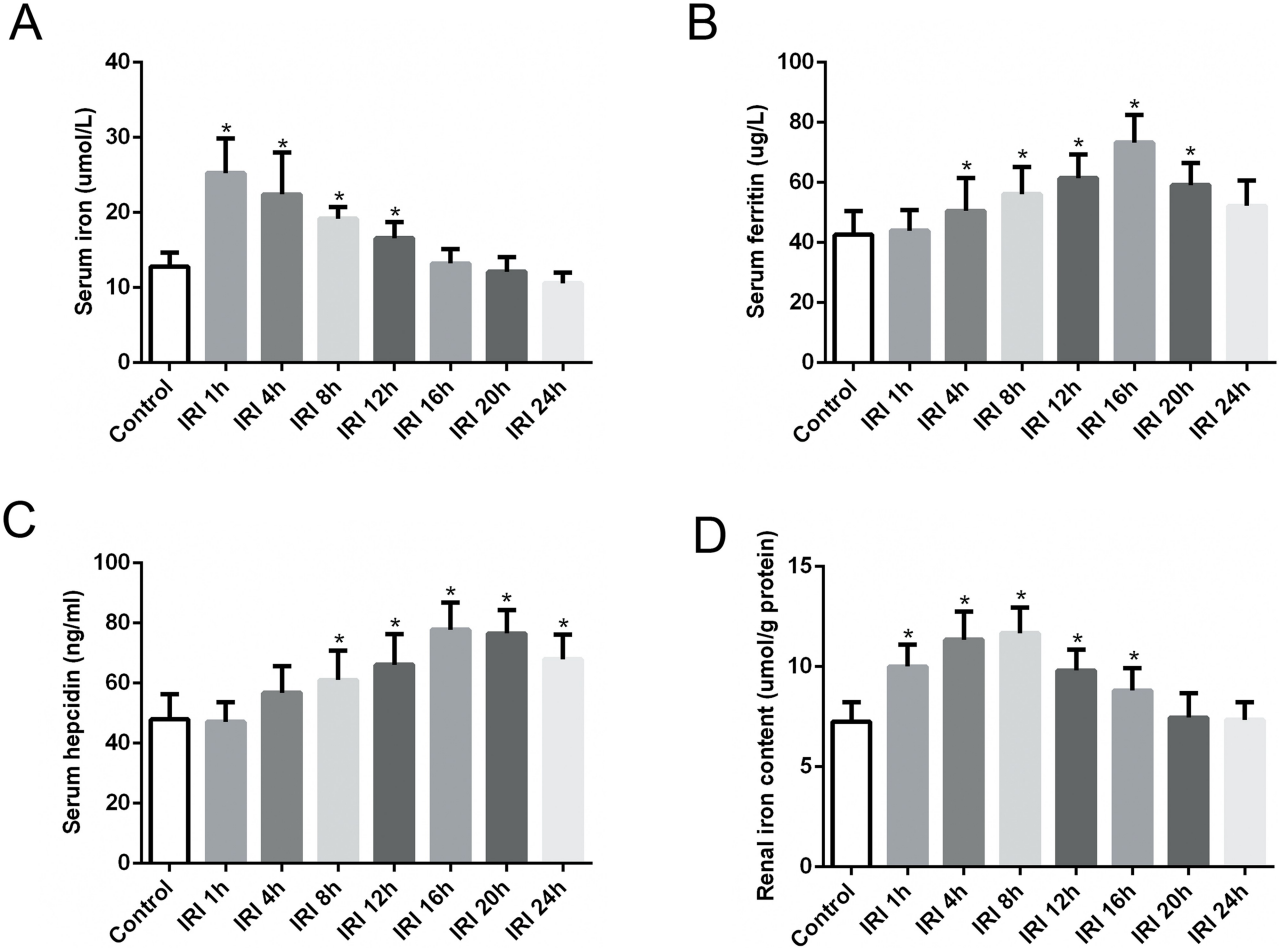
Evaluation of iron metabolism indexes in the serum and kidney after renal IRI. (A) SI increased significantly early after reperfusion, and it time-dependently decreased after reperfusion in the IRI groups. (B) The level of SF began to rise at 4 h after reperfusion and started to decline after reaching a maximum value at 16 h after reperfusion in the IRI groups. (C) Serum hepcidin increased significantly beginning at 8 h after reperfusion and started to decline after reaching a maximum value at 16 h after reperfusion in the IRI groups. (D) Renal iron content was time-dependently increased from 1 h to 8 h after reperfusion and then declined gradually in the IRI groups. The data are presented as the means ± standard deviation. * P <0.05, compared to control group; n = 6 in each group. IRI: ischemia/reperfusion injury; SI: serum iron; SF: serum ferritin.

We found that SI increased significantly early after reperfusion, and it was time-dependently decreased after reperfusion in the IRI groups. In addition, SI was significantly elevated in the IRI 1 h, IRI 4 h, IRI 8 h and IRI 12 h groups compared with levels in the control group. However, there were no significant differences between the IRI 16 h, IRI 20 h, IRI 24 h groups and the control group (Fig 2A).

The level of SF began to rise 4 h after reperfusion and started to decline after reaching a maximum value at 16 h after reperfusion in the IRI groups. SF levels in the IRI 4 h, IRI 8 h, IRI 12 h, IRI 16 h and IRI 20 h groups were significantly elevated compared with levels in the control group. However, SF levels in the IRI 1 h and IRI 24 h groups were not significantly different than the SF level in the control group (Fig 2B).

As shown in Fig 2C, the serum hepcidin concentration began to rise 4 h after reperfusion in the IRI groups, but there was no significant difference before 4 h compared with the concentration in the control group. Serum hepcidin levels increased significantly beginning at 8 h after reperfusion and started to decline after reaching a maximum value at 16 h after reperfusion in the IRI groups. Nevertheless, the serum hepcidin levels at 24 h after reperfusion in the IRI groups were still significantly higher than that of the control group.

### Evaluation of iron content in the kidneys, spleen, liver and duodenum after renal IRI

**Fig 3:**
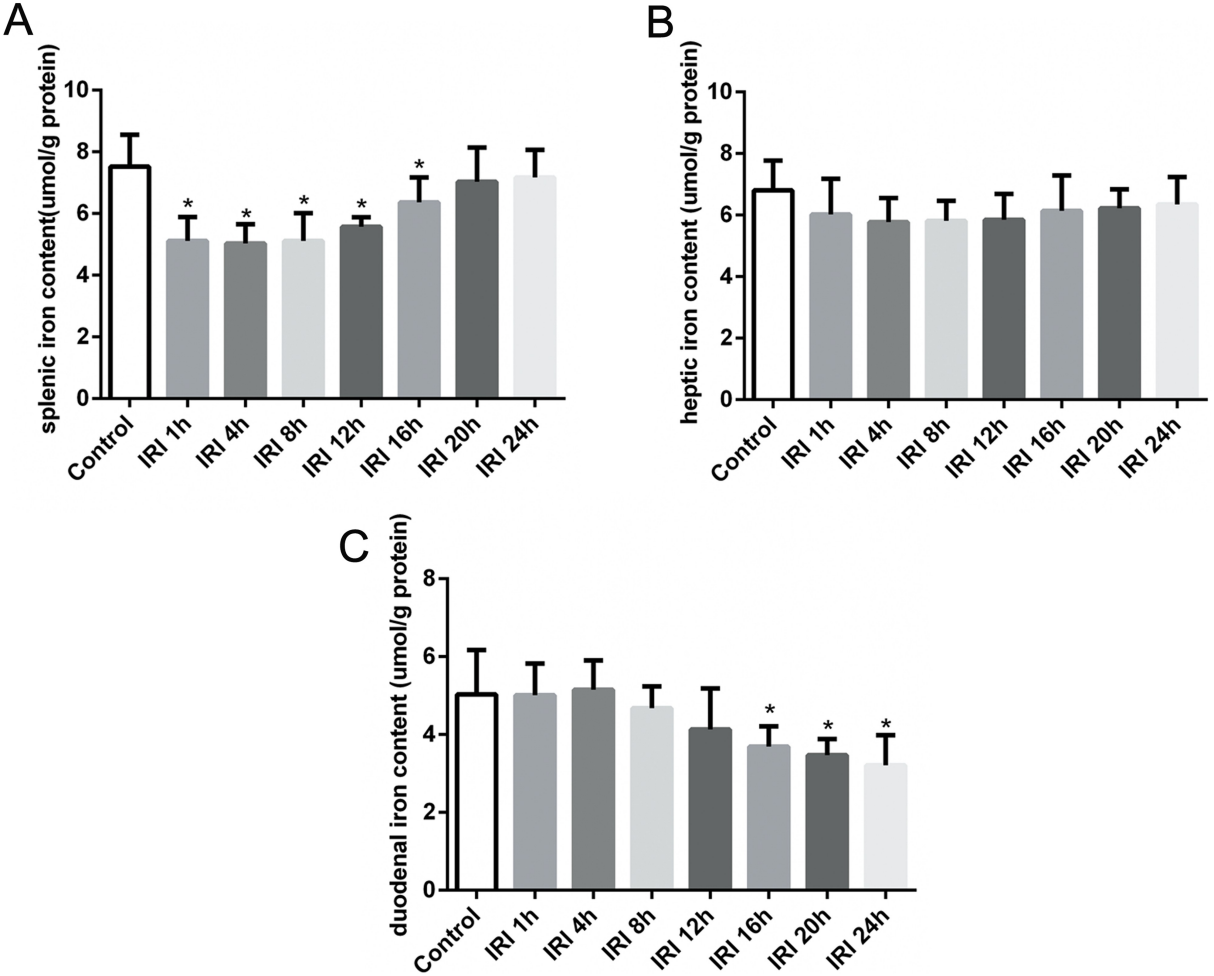
Iron analysis in the spleen, liver and duodenum after IRI. (A) The splenic iron content was significantly decreased in the early stage after IR and then increased time-dependently with increasing reperfusion time. (B) The hepatic iron content declined in the early stage of IRI but was not significantly different than the hepatic iron content in the control group. (C) The duodenal iron content showed no obvious change in the early stage after reperfusion, but it time-dependently decreased since 16 h after reperfusion in the IRI groups. The data are presented as the means ± standard deviation. * P <0.05, compared to control group; n = 6 in each group. IRI: ischemia/reperfusion injury.

We assessed renal iron content to analyze iron metabolism in the kidneys after renal reperfusion. Fig 2D shows that renal iron content time-dependently increased from 1 h to 8 h after reperfusion and then declined gradually in the IRI groups. In addition, compared with levels in the control group, renal iron levels were significantly elevated in the IRI 1 h, IRI 4 h, IRI 8 h, IRI 12 h and IRI 16 h groups. The splenic iron content decreased significantly in the early stage after IR and then increased time-dependently with increasing reperfusion time, but there was no significant difference in the IRI 20 h and IRI 24 h groups compare to the content in the control group (Fig 3A). The hepatic iron content declined in the early stage of IRI but was not statistically different compared to the content in the control group after reperfusion (Fig 3B). The duodenal iron content showed no obvious change in the early stage after reperfusion, but it time-dependently decreased since 12 h after reperfusion in the IRI groups, and it was significantly decreased in the IRI 16 h, IRI 20 h and IRI 24 h groups compared to the content in the control group (Fig 3C).

### RT-PCR analyses of hepcidin and FPN1 after renal IRI

**Fig 4:**
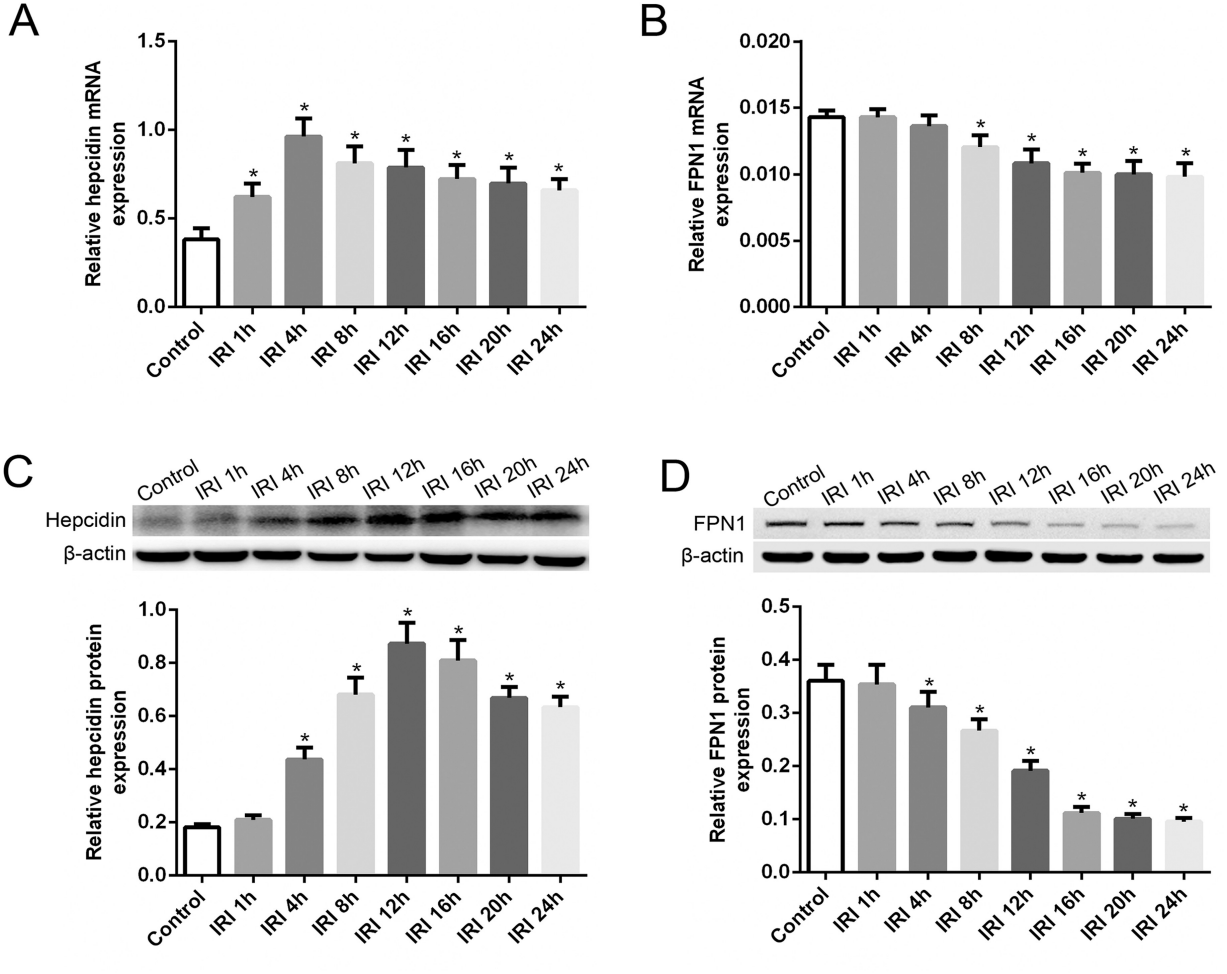
Liver hepcidin expression and kidney FPN1 expression after IRI. (A) In the IRI groups, hepcidin mRNA levels increased rapidly after reperfusion and then declined gradually beginning at 8 h after reperfusion. (B) FPN1 mRNA levels in the kidney decreased gradually after reperfusion in the IRI groups. (C) Hepcidin protein expression was significantly increased beginning at 4 h after reperfusion and started to decline after reaching a maximum value at 12 h after reperfusion in the IRI groups. (D) FPN1 protein expression in the kidney clearly decreased beginning at 4 h after reperfusion in the IRI groups compared with expression in the control group. The data are presented as the means ± standard deviation. * P <0.05, compared to control group; n = 6 in each group. FPN1: ferroportin-1; IRI: ischemia/reperfusion injury.

Based on the RT-PCR analysis, we found that hepcidin mRNA levels in the liver were significantly increased in the IRI groups compared with levels in the control group. In the IRI groups, hepcidin mRNA levels increased rapidly after reperfusion and then gradually declined beginning at 8 h after reperfusion; however, the levels in the IRI groups were still much higher at 24 h after reperfusion than the level in the control group (Fig 4A). In contrast, in the IRI groups, renal FPN1 mRNA levels decreased gradually after reperfusion and decreased significantly beginning at 8 h after reperfusion compared with levels in the control group. Nevertheless, the trend toward decreasing FPN1 mRNA levels almost stopped beginning at 12 h after reperfusion in the IRI groups (Fig 4B).

### Western blot analyses of hepcidin and FPN1 after renal IRI

We further confirmed the increase in hepcidin protein levels in the liver and the reduction in FPN1 protein levels in the kidneys via western blot analysis. Hepcidin protein expression was significantly increased beginning at 4 h after reperfusion and started to decline after reaching a maximum value at 12 h after reperfusion in the IRI groups; however, it was still significantly higher at 24 h after reperfusion than in the control group (Fig 4C). FPN1 protein expression in the kidneys clearly decreased beginning at 4 h after reperfusion in the IRI groups compared with expression in the control group, and it almost stopped decreasing and remained at a low level beginning at 16 h after reperfusion (Fig 4D).

### Immunohistochemical analyses of hepcidin and FPN1 after renal IRI

**Fig 5:**
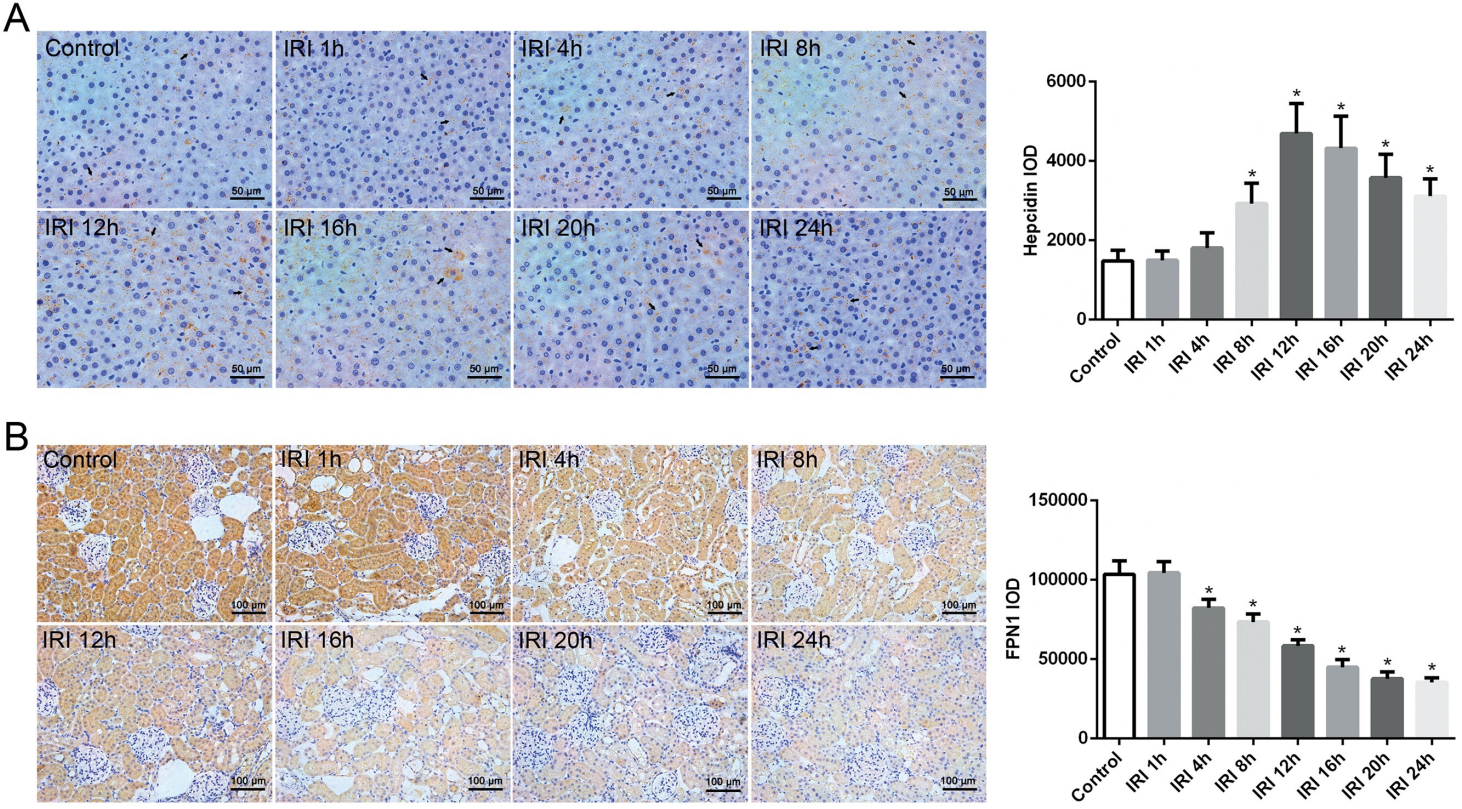
Immunohistochemical analyses of hepcidin and FPN1 after renal IRI. (A) Hepcidin protein expression in the liver increased gradually after renal reperfusion. Arrow denotes hepcidin in the hepatocytes. (B) FPN1 protein expression in the kidney clearly decreased after reperfusion. Original magnification of liver tissues, X400. Original magnification of kidney tissues, X200. The data are presented as the means ± standard deviation. * P <0.05, compared to control group; n = 6 in each group. FPN1: ferroportin-1; IRI: ischemia/reperfusion injury; IOD: integrated optical density.

We calculated the integrated optical density (IOD) of immunohistochemical staining to analyze the expression levels of hepcidin in the liver and FPN1 in the kidneys. The results show that hepcidin expression in the liver increased gradually after renal reperfusion, and it increased significantly beginning at 8 h compared with expression in the control group (Fig 5A). Along with the increase in hepcidin, FPN1 expression in the kidneys clearly decreased after reperfusion (Fig 5B).

## Discussion

Kidneys are highly perfused organs that function in iron filtration, reabsorption and excretion, but they are susceptible to ischemia. Renal IRI refers to the pathological phenomenon of renal tissue and cell damage that occurs during ischemia. After blood reperfusion of kidneys, tissue injury did not show improvement but was further aggravated. The early detection of renal IRI and initiation of treatment might help prevent damage due to IRI or might improve renal function after IRI.

Animal models of renal IRI have been used as tools to study the pathophysiological mechanisms of this condition and to test the efficacy of different therapies [13, 15, 16]. The renal IRI model involves the occlusion of renal blood flow using a non-traumatic vascular clamp applied to the renal pedicle or renal artery, leading to renal ischemia. After an appropriate amount of time, the clamp is removed to allow blood reperfusion. This blood occlusion and reperfusion leads to renal injury, namely, renal IRI.

The results of the present study confirmed that the renal blood occlusion and reperfusion model induced renal injury, as shown by varying degrees of renal pathological changes from 1 h to 24 h after reperfusion in the IRI rats, including the loss of brush borders, vacuolar degeneration and necrosis in epithelial cells, and dilatation, cast formation, and cell debris in the kidney tubules (Fig 1A). In addition, as previously described for this model [15, 16], renal insufficiency existed in the IRI rats, as shown by the significantly increased SCr and BUN concentrations (Fig 1B and 1C). These values were time-dependently increased from 1 h to 24 h after reperfusion. The results reveal that, although renal ischemia lasted for only a short period of time and was followed immediately by blood reperfusion, the pathological changes and renal insufficiency were not improved but were further exacerbated.

Iron is known to play an important role in oxidative tissue injury through its ability to catalyze the production of reactive oxide species (ROS) via the Fenton reaction [17]. Previous studies revealed that iron-related oxidative stress-induced apoptotic cell death is one of the major pathways involved in the pathogenesis of renal IRI [3, 18, 19]. Iron overload can worsen kidney injury. Additionally, iron chelation and antioxidants attenuate renal IRI [20, 21], providing indirect evidence for iron metabolism dysfunction. In the present study, we observed regular variations in the iron metabolism indexes in the blood and kidneys in renal IRI. SI was significantly and rapidly increased after IR compared with levels in the control group. SI then decreased slowly with increasing reperfusion time (Fig 2A). The SF concentration, which reflects the state of iron in the blood, was significantly increased in IRI rats from 4 h to 20 h after IR compared with levels in the control (Fig 2B). In addition, the renal iron content significantly increased, accompanied by renal pathological injury, from 1 h to 16 h in IRI rats compared to levels in the control (Fig 2D). Hypoxia/reoxygenation is known to enhance the production of free radicals [22], the increased renal iron content might enhance the renal pathological injury in renal IRI. These findings in our study demonstrate an iron metabolism disorder in IRI, which likely contributes to kidney injury in IRI.

Hepcidin, a small peptide synthesized in the liver, is a key regulator of iron absorption and homeostasis in mammals [23]. In recent years, the mechanisms underlying hepcidin and iron regulation have been frequently studied. Liver hepcidin expression is regulated by the balance between several factors, including iron levels, hypoxia and anemia [24]. Hypoxia and anemia suppress liver hepcidin expression via hypoxia-inducible transcription factors (HIFs) to regulate iron homeostasis [25, 26]. In contrast, iron could regulate hepcidin expression. Previous studies have shown that iron overload can up-regulate hepcidin expression, and iron deficiency can down-regulate its expression, which is an important negative feedback mechanism for iron homeostasis in the body [27, 28]. Recent studies have provided further evidence that iron is the key regulator of hepcidin gene expression via the BMP6/SMAD signaling pathway in hepatocytes [14, 29, 30]. Accordingly, we observed a significant increase in SI levels (Fig 2A) and liver hepcidin gene overexpression (Fig 4A) compared with observations in the control rats. Our data show that SI significantly increased early after reperfusion, and liver hepcidin mRNA expression was almost simultaneously rapidly elevated. With the extension of reperfusion time, the SI level declined gradually, and the liver hepcidin mRNA expression level showed the same tendency. These results suggest that the increase in SI may contribute to hepcidin overexpression in the liver in IRI.

However, the reason for the increase in SI in IRI is unclear. The liver and spleen are known to be the main storage sites and sources of iron in the body [31]. Previous studies have indicated that they play an important role in the pathophysiology of renal IRI [32, 33]. To verify the hypothesis that whether the increase in SI is associated with iron storage organs, we evaluated the iron content of these organs, as well as the iron content of the duodenum, which can express FPN1 and divalent metal transporter 1, both of which are involved in iron absorption from food and the transfer of iron to the circulation [34, 35]. We found that the splenic iron content was significantly decreased in the early stage after IR, and it then increased time-dependently with increasing reperfusion time (Fig 3A). The hepatic iron content declined in the early stage of IRI but was not significantly different than the hepatic iron content in the control group (Fig 3B). The duodenal iron content showed no obvious change in the early stage after reperfusion (Fig 3C). These results indicated that the spleen and liver might contribute to the increase in SI in the early stage of IRI. The lack of a significant decline in hepatic iron content in the early stage of IRI may be due to the extensive iron storage capacity of the liver, where the release of some iron may not result in a significant change in renal IRI.

Previous studies indicate hepcidin function in iron intake of the reticuloendothelium; besides, the splenic macrophages, which act as the central role in maintaining body iron homeostasis, respond more acutely to a hepcidin change than other organs [36, 37]. Accordingly, we observed a time-dependently increase of the splenic iron content in later period after reperfusion in the IRI groups, accompany with the increase of hepcidin. The dramatic change in splenic iron content might suggest its crucial contribution to the change of SI in this model. Additionally, duodenal iron content showed a time-dependent downtrend since 12 h after reperfusion, and it was significantly decreased in the later period after reperfusion in the IRI groups compared to the content in the control group, which might lessen iron exportation form duodenum to the circulation, and contribute to the decrease of SI in this model. The change of the iron content in duodenum might result from the elevated hepcidin in IRI. This phenomenon consistent with previous studies, which have reported inhibited iron uptake and iron efflux in intestines following exposure to increased of hepcidin [38, 39]. However, our findings were not completely in accordance with the findings of Swaminathan [40], who observed an obvious decrease in the iron content in the liver and spleen, as well as significantly higher SI levels, at 24 h following IR. The differences in these results might be due to differences in IRI models or differences in the length of renal occlusion (24–26 min), which might ultimately result in a different reaction of the body.

FPN1 is an iron export transmembrane protein that is widely distributed throughout the body and is presently the only known mechanism for the release of iron from cells [41, 42]. In the kidneys, the FPN1 protein is primarily located in the proximal tubule and in part of the medullary renal tubule [43, 44].With the assistance of the FPN1 accessory protein, filtered iron can reenter blood circulation via FPN1 [45]. In the present study, we observed a significant decrease in FPN1 expression in the kidneys after reperfusion in the IRI rats via western blot analysis (Fig 4D) and immunohistochemistry (Fig 5B), along with an increase in renal iron content. In contrast, hepcidin was found to be increased in the liver and serum of IRI rats. The primary known function of hepcidin is to induce FPN1 degradation and increase intracellular iron stores [8, 23]. However, whether the decrease in FPN1 protein levels in the kidneys were mainly due to the high levels of hepcidin or were also a result of the decreased FPN1 mRNA expression levels, which showed unparallel decline with FPN1 protein expression after reperfusion in IRI groups, is unclear. In mammals, FPN1 plays a critical role in both systemic and cellular iron balance, it is regulated by iron regulatory hormone hepcidin, and the interaction of iron regulatory proteins (IRPs) and iron responsive elements (IREs) [34, 41, 46, 47]. Cellular iron homeostasis is coordinately regulated by iron IRP1 and IRP 2, which posttranscriptionally modulate the expression of critical iron metabolism genes, including transferring receptor 1 and FPN1 by interacting with IREs present in the untranslated region (UTR) mRNAs [47]. Additionally, it is reported that cellular hypoxia can stimulate the expression of IRPs, the IRP-mediated regulation of FPN1 and other iron metabolism proteins leads to iron accumulation, and dysregulation of these IRPs in hypoxia/reoxygenation is responsible for pathology of tissue injury [48]. The accurate mechanism results in the down-regulation of FPN1 expression in renal IRI needs further studies. On all accounts, the decline in FPN1 protein expression might benefit iron sequestration of tubular cells to reduce the release of catalytic iron and attenuate the toxic effects on the kidneys.

In summary, the features of the iron metabolism indexes were observed dynamically within 24 h in a renal IRI model. We found that this model of renal IRI presented worsening renal dysfunction and renal lesions within 24 h after reperfusion. The increase in SI and renal iron content might contribute to the development of renal lesions via iron-dependent oxidant injury. The early increase in SI might result from the release of iron by the spleen and liver. Our data also suggest that the increase in SI led to hepcidin overexpression in the liver. The increased hepcidin secretion would facilitate iron sequestration by prompting the intake of iron by the spleen and liver and restricting iron intake and exportation by the duodenum. Besides, hepcidin might participate in down-regulating FPN1 expression in renal tubular cells to attenuate iron-dependent oxidant injury in renal IRI. The changes in iron indexes in our study demonstrate an iron metabolism disorder in renal IRI, and hepcidin might be involved in maintaining iron homeostasis in renal IRI. These findings indicate that there might be a self-protection mechanism regulating iron homeostasis in renal IRI and provide a new perspective on iron metabolism in attenuating renal IRI.
